# Adopting a systems-thinking approach to optimise dietary and exercise referral practices for cancer survivors

**DOI:** 10.1007/s00520-024-08692-z

**Published:** 2024-07-10

**Authors:** Ria Joseph, Nicolas H. Hart, Natalie Bradford, Fiona Crawford-Williams, Matthew P. Wallen, Reegan Knowles, Chad Y. Han, Vivienne Milch, Justin J. Holland, Raymond J. Chan

**Affiliations:** 1 Caring Futures Institute, College of Nursing and Health Sciences, Flinders University, Adelaide, SA Australia; 2 Faculty of Health, Human Performance Research Centre, INSIGHT Research Institute, University of Technology Sydney (UTS), Sydney, NSW Australia; 3 Exercise Medicine Research Institute, School of Medical and Health Sciences, Edith Cowan University, Perth, WA Australia; 4 Institute for Health Research, The University of Notre Dame Australia, Perth, WA Australia; 5 Cancer and Palliative Care Outcomes Centre, School of Nursing, Queensland University of Technology, Brisbane, QLD Australia; 6 School of Science, Psychology and Sport, Federation University Australia, Ballarat, VIC Australia; 7 Cancer Australia, Sydney, NSW Australia; 8 The University of Notre Dame, Sydney, NSW Australia; 9 School of Exercise and Nutrition Sciences, Queensland University of Technology, Brisbane, QLD Australia; 10 Division of Cancer Services, Princess Alexandra Hospital, Metro South Health, Brisbane, QLD Australia

**Keywords:** Cancer care, Diet, Exercise, Referral practices, Health system, Systems-thinking

## Abstract

**Purpose:**

Service referrals are required for cancer survivors to access specialist dietary and exercise support. Many system-level factors influence referral practices within the healthcare system. Hence, the aim of this study was to identify system-level factors and their interconnectedness, as well as strategies for optimising dietary and exercise referral practices in Australia.

**Methods:**

A full-day workshop involving national multidisciplinary key stakeholders explored system-level factors impacting dietary and exercise referral practices. Facilitated group discussions using the nominal group technique identified barriers and facilitators to referral practices based on the six World Health Organisation (WHO) building blocks. The systems-thinking approach generated six cognitive maps, each representing a building block. A causal loop diagram was developed to visualise factors that influence referral practices. Additionally, each group identified their top five strategies by leveraging facilitators and addressing barriers relevant to their WHO building block.

**Results:**

Twenty-seven stakeholders participated in the workshop, including consumers (*n* = 2), cancer specialists (*n* = 4), nursing (*n* = 6) and allied health professionals (*n* = 10), and researchers, representatives of peak bodies, not-for-profit organisations, and government agencies (*n* = 5). Common system-level factors impacting on referral practices included funding, accessibility, knowledge and education, workforce capacity, and infrastructure. Fifteen system-level strategies were identified to improve referral practices.

**Conclusion:**

This study identified system-level factors and strategies that can be applied to policy planning and practice in Australia.

**Supplementary Information:**

The online version contains supplementary material available at 10.1007/s00520-024-08692-z.

## Introduction

Increasing evidence shows that obesity, cardiovascular disease, and metabolic syndrome negatively impact the overall health, physical function, and quality of life of cancer survivors [1]. Other conditions including malnutrition, cachexia, sarcopenia, and reductions in bone mineral density can all occur as late effects of cancer and its treatment. Dietary and exercise interventions for cancer survivors can prevent, attenuate, or reverse multiple adverse physical and psychosocial effects of cancer and its treatment, through their impact on other coexisting chronic medical conditions, such as cardiovascular disease or obesity [2, 3]. As such, it is critical that routine dietary and exercise interventions are incorporated into standard cancer care.

For the majority of cancer survivors, a targeted, coordinated multidisciplinary approach incorporating medical, nursing, and allied health professionals (i.e. dietitians and exercise professionals) is recommended to maximise improvements in nutritional status, physical functioning, and overall quality of life [4–6]. It is important to recognise that cancer care professionals have different roles and responsibilities in terms of providing dietary and exercise support to cancer survivors. While medical and nursing health professionals play an important role in communicating the benefits of improving diet and participating in exercise to cancer survivors and reinforcing positive behaviour change, they require additional support to provide specialised recommendations for cancer survivors depending on the survivor’s clinical and sociodemographic situation [7, 8]. In such circumstances, medical and nursing health professionals’ roles may include facilitating referrals to dietitians and exercise professionals (i.e. clinical exercise physiologists and physiotherapists) for individualised dietary and exercise support to meet the complex needs of cancer survivors [8]. Dietary interventions should be designed and delivered by dietitians to facilitate the provision of individualised nutritional plans that improve dietary intake and alleviate nutrition-related side effects associated with cancer and its treatment [9]. Similarly, exercise interventions should be designed and delivered by qualified exercise professionals who can prescribe safe and effective exercise programmes that increase cardiorespiratory fitness and physical function; improve body composition, psychosocial wellbeing, and quality of life; and promote cancer recovery [10].

In the current context, cancer survivors may or may not receive dietary and exercise advice from medical (primary care and specialist oncology care), nursing, and allied health professionals as part of their routine cancer care [8]. For cancer survivors to access specialised dietary and exercise care [11] in Australia, they may need to be initially referred to dietitians and exercise professionals by their specialist team or general practitioner (GP). If referred by a GP, cancer survivors can access subsidised dietary and exercise services via chronic disease management plans through universal healthcare; however, there are limits and caveats to this access [12]. Cancer survivors may be referred by their specialist team; however, around half (53%) of the public and private hospitals in Australia that provide cancer care do not have established referral pathways for supportive care services, with only 19% of hospitals referring cancer survivors to external organisations or allied health professionals [13]. Referral rates are similar in the UK and the US where referrals occur less frequently due to several barriers experienced by medical, nursing, and allied health professionals [14, 15]. This highlights a gap with current referral practices, the importance of standardising referral processes, and issues related to supportive care access for cancer survivors [13].

In order to address similar concerns in the provision of dietary and exercise support, previous approaches include raising awareness among cancer survivors and their healthcare professionals about the benefits of diet and exercise, engaging stakeholders for their buy-in within different clinical organisations, implementing validated triage tools, and integrating referrals into the survivorship care plan and/or electronic records [15–17]. Consensus for the essential elements of diet and exercise referral practices has also been developed [18]. However, optimising referral practices requires a better understanding of complex factors that are part of an interconnected system that may facilitate or impede referral practices. To our knowledge, only factors at an individual level have been investigated [7, 19–21]. Limited research has examined these factors at a system level, how they are interconnected and how to devise innovative strategies that target them effectively.

Systems thinking involves the exploration of characteristics and components within a system through a holistic and complex lens, focusing on how components of the system are interconnected and how they interact in complex ways, to improve understanding of how healthcare outcomes may emerge from these interactions [22–24]. The World Health Organisation’s (WHO) building blocks categorise health systems in terms of six building blocks: (1) financing, (2) health workforce, (3) information, (4) technologies, (5) leadership and governance, and (6) service delivery [25]. These building blocks provide a conceptual framework for identifying system-level factors and relationships between factors that interact in ways that may influence dietary and exercise referral practices in a cancer setting. Many system-level factors influence the quality and effectiveness of existing referral practices within the healthcare system in Australia. Understanding how those factors are interconnected, and leveraging existing strategies and promoting new synergies between them, can help to enhance facilitators and overcome barriers to referral.

The aim of this study was to pioneer a systems-thinking approach using the WHO building blocks to (1) identify relevant system-level factors related to dietary and exercise advice and referral practices, (2) understand the interactions between factors across different building blocks of the healthcare system, and (3) identify innovative strategies that leverage existing synergies and create or promote new relationships between various system-level factors, ultimately optimising dietary and exercise advice and referral practices.

## Materials and methods

### Study design

A systems-thinking approach was used to explore dietary and exercise advice and referral practices, framed by the WHO health system building blocks framework [26], and was facilitated through a workshop with groups of key multidisciplinary stakeholders. Stakeholders were recruited from different healthcare settings and included cancer consumers/consumer representatives and multidisciplinary care providers, including primary care, oncology specialists, dietitians, exercise professionals, researchers, and representatives of peak bodies (non-governmental organisations whose membership consists of smaller organisations of allied interests) such as Dietitians Australia (DA) and Exercise & Sports Science Australia (ESSA), Cancer Council (not-for-profit organisation), and Cancer Australia (the Australian Government agency for cancer control). A group of diverse stakeholders were recruited to ensure that critical perspectives relevant to each of the six WHO building blocks were represented at the workshop.

Systems-thinking approaches have been used frequently in public policy [27]. Although its application in health settings is limited, it has been used to map and address complex problems in obesity and diabetes [28]. Cognitive mapping was used to explore the characteristics of and interactions within the Australian healthcare system that may impact dietary and exercise referral practices and was subsequently mapped to the WHO building blocks framework. This technique creates a visual representation of a group’s findings for a process or concept, such as illustrating the relationships between identified factors [29]. When relationships were identified, cognitive maps were consolidated into a causal loop diagram to describe a set of interlinked feedback loops representing the processes involved in implementing healthcare system changes [30]. Ethics approval was provided by the Human Research Ethics Committee of Flinders University (HREC ID: 5566). All stakeholders provided written consent prior to any participation in this study. Data were collected and managed in accordance with the World Medical Association’s Declaration of Helsinki.

### Part 1: Pre-workshop preparation

Potential stakeholders were identified and invited to participate via existing networks of the research team. Prior to the workshop, participant demographics were obtained via an online questionnaire, including gender, age, location of occupation, current profession, and time (in years) working in cancer care (if applicable). A workshop booklet was also distributed, compromising of a workshop outline and background information on the (1) workshop methodology, (2) relevant material relating to the essential elements of dietary and exercise referral practices [8], and (3) the WHO health system building blocks framework (Online Resource 1). Participants were pre-assigned to a group discussing one of the six WHO building blocks by the research team based on their previous experiences, knowledge, and expertise in those areas. Facilitators of each group were tasked with (1) reviewing the Principles of Cancer Survivorship [31] and essential elements of dietary and exercise referral practices [18] and (2) familiarising themselves with a semi-structured workshop guide to promote discussions within the groups as they apply to their group’s allocated WHO building block throughout each session.

### Part 2: Workshop

A full-day systems-thinking workshop using the nominal group technique was held face-to-face with key stakeholders at a South Australian University. Prior to the start of the discussion, participants were provided with an overview of the topics as well as step-by-step instructions for each of the sessions listed below (approximately 30 min). Participants were directed to their pre-assigned groups to discuss one of the six WHO building blocks. All discussions were audio-recorded by facilitators and transcribed verbatim by the research team for analysis.

The workshop was divided into three sessions:

1. Identification of contextual factors in the healthcare system related to dietary and exercise referral practices (approximately 60 min)
2. Discussion of the relationships between the factors across the WHO health system building blocks (approximately 100 min)
3. Identification and discussion of innovative strategies that may address the identified system-level barriers to foster dietary and exercise referral practices for cancer survivors (approximately 150 min).

#### Session 1: System-level factors

Each group was involved in small-group discussions on the functions of the healthcare system and identifying system-level factors impacting referral practices related to their allocated WHO building block. Participants used individual sticky notes for each identified factor to develop a group cognitive map written on paper, highlighting factors that may influence dietary and exercise referral practices for cancer survivors. Factors that were not considered relevant to the topic were still documented and refined during data analyses.

#### Session 2: Interactions between WHO building blocks

This session was divided into two parts. Firstly, small-group discussions within each group explored how the allocated WHO building block interacted with the other building blocks and identified relationships between them. Participants were asked to expand their cognitive maps by drawing relationships between the system-level factors using markers. Relationships were defined as causal links between two variables (e.g. if a change in the level of one variable causes a change in the other variable either positively or negatively). The strength of relationships between factors was not considered. Secondly, a facilitator from each small group summarised key discussion points and presented their cognitive maps, including the identified relationships, to the full stakeholder group.

#### Session 3: Innovative strategies

The third session was divided into two parts. Firstly, small-group discussions identified and summarised innovative strategies that would leverage system-level facilitators, address system-level barriers, and address causal relationships between these factors to optimise dietary and exercise referral practices for cancer survivors. Participants were encouraged to identify one or more strategies to address each of the key barriers relevant to their WHO building block. For the purpose of this session, the groups discussing the information and technology building blocks were combined due to the similar nature of these discussions. Secondly, the facilitator from each small group summarised and presented their group’s top five strategies to overcome system-level barriers within their WHO building block to the full stakeholder group for each WHO building block to enable further large group discussion.

### Data analyses

Participant demographics were descriptively analysed and reported, and count data were expressed as *n* (%). A deductive thematic approach was used to analyse qualitative data from the systems-thinking workshop. Data were categorised under the WHO health system building blocks framework. Common themes that emerged across the six WHO building blocks were grouped into categories. Strategies that were identified in the third session were tabulated and refined to avoid duplication.

### Causal loop diagram development

Causal loop diagrams have been highly effective in bringing key stakeholders together to address common healthcare concerns and yet remain underutilised in this regard [32–35]. This study used a three-step process to develop and validate a causal loop diagram as described below: (1) data collection to inform the development of the causal loop diagram, (2) the development of the causal loop diagram, and (3) validation of the causal loop diagram.

#### Step 1: Data collection

Vensim (Ventana Systems, Inc.; Harvard, USA) [36], a computer software package specific for system dynamics, was used to consolidate the cognitive maps into a causal loop diagram with reference to written notes and audio-recordings. This ensured that all relationships discussed were accurately reflected in the diagram. Vensim has been used in previous studies exploring a systems approach to healthcare for developing causal loop diagrams [34, 37].

#### Step 2: Development of causal loop diagram

**Fig. 1:**
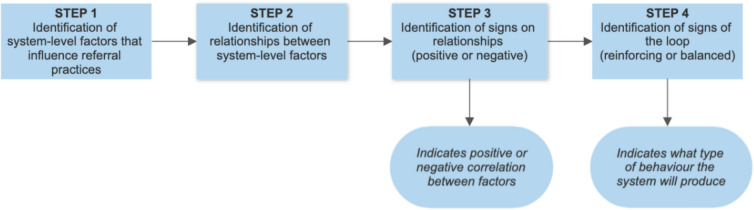
Development of causal loop diagram

Once the factors were identified, Vensim linked factors together and identified the direction of the relationships (Fig. 1). One-headed arrows were drawn between factors highlighting these relationships, as well as indicating the causal direction of the perceived causal effect. If factor A moved in the same direction as factor B, the link from factor A to B was labelled with a ‘ + ’; if factor B changed in a direction opposite of factor A (i.e. as A increases, B decreases), the link from A to B was labelled with ‘-’. Once the links were completed, the type of behaviour it produced was then determined. A series of arrows that close to form a loop were labelled as either a reinforcing or balanced loop. The dynamics of any system stem from the interaction of two types of feedback loops: reinforcing and balanced loops. Specifically, reinforcing loops tend to amplify whatever is happening in the system, whereas balanced loops counteract and oppose the change [38]. To determine whether a causal loop was reinforcing or balancing, the number of ‘-’s was counted. If there was an even number of ‘-’s (or none present), the loop was reinforcing and was labelled ‘R’. If there was an odd number of ‘-’s, it was a balancing loop and was labelled ‘B’ [39].

#### Step 3: Validation of the causal loop diagram

Validation involved refining the draft causal loop diagram through meetings with the research team. The group discussed the variables, the nature of the relationships, and the overall structure of the causal loop diagram. The causal loop diagram was refined based on this feedback. After the development of the causal loop diagram, identified strategies were linked with corresponding loops in the causal loop diagram.

## Results

Twenty-seven stakeholders participated in the systems-thinking workshop. Table 1 presents the participant demographics. Participants were predominantly based in South Australia (44%) and Queensland (30%) and were mainly nursing professionals (22%), dietitians (19%), exercise professionals (19%), medical practitioners (15%), and consumers (7%). Of these, participants were primarily employed with dual roles as dietitians/researchers (19%) and exercise professionals/researchers (11%). The median years of experience in cancer care were 8.67 years.

**Table 1:** Demographic characteristics of workshop participants

Characteristics (*n* = 27)	Number (*n*)	Percent (%)
**Sex**		
Female	17	63
Male	9	33
Prefer not to say	1	4
**Age group**		
25–34	6	22
35–44	11	41
45–54	4	15
55–64	3	11
65 or older	2	7
Prefer not to say	1	4
**Ethnicity**		
Caucasian	20	74
Asian	4	15
Other	2	7
British Australian	1	4
British	1	4
Prefer not to say	1	4
**State/territory**		
Australian Capital Territory	1	4
New South Wales	3	11
Queensland	8	30
South Australia	12	44
Victoria	3	11
Northern Territory/Tasmania/Western Australia	0	0
**Profession**		
Consumer	2	7
Consumer/researcher**	1	4
Consumer involvement in research**	1	4
Dietitian	5	19
Dietitian/researcher**	5	19
Exercise professional	5	19
Exercise physiologist	1	4
Physiotherapist	1	4
Exercise professional/researcher**	3	11
Medical practitioner	4	15
Medical practitioner/researcher**	1	4
Nursing professional	6	22
Nurse	3	11
Nurse practitioner	1	4
Nurse professional/researcher**	2	7
Researcher	3	11
Other	2	7
Policy advocate	1	4
CEO NFP Patient Support Organisation*	1	4
**Cancer care experience (years)**		
0 to 4	8	30
5 to 9	6	22
10 to 14	7	26
15 to 19	4	15
20 or more	2	7

^*^Abbreviations: *CEO NFP* chief executive officer of non-for-profit organisation

^**^Participants with dual roles

The views of participants on system-level factors influencing dietary and exercise referral practices for cancer survivors were grouped into six main categories as per the WHO building blocks (Online Resource 1).

### Session 1: System-level factors

Several barriers (Online Resource 2) were identified by participants as hindering access to dietary and exercise services within each of the WHO health system building blocks. These included barrier pertaining to financing (i.e. lack of funding, out-of-pocket costs for patients, and resource allocation), service delivery (i.e. infrequent and inconsistent screening practices, inadequate use of guidelines and standards, and insufficient allied health sessions through the chronic disease management plan), health information (i.e. lack of training and continuing professional development, conflict role identity of health professionals, lack of awareness of resources and services, and lack of digital and health literacy), leadership/governance (i.e. fragmented leadership and responsibilities, lack of care coordination, and involvement of all stakeholders from the beginning resulting in a fragmented system), workforce (i.e. limited staff capacity and services, time constraints, and patient demand), and technology (i.e. lack of communication pathways between health professionals and patients, lack of connections between information systems, and technologies not being supported by healthcare systems).

### Session 2: Interactions between WHO building blocks

Six cognitive maps (Online Resource 3) were developed by participants, highlighting the various relationships identified between factors. These included relationships between funding and resource utilisation, resource allocation, workforce capacity, health education, digital and health literacy, digital health communication, and screening and referral practices.

### Session 3: Innovative strategies

In considering the numerous system-level barriers (Online Resource 2) identified by workshop participants, 15 respective strategies were identified across the small groups that can be used to address some of the key barriers discussed. These strategies can be used to further advance practices in health professionals’ guidance and referrals for dietary and exercise services (Table 2).

**Table 2:** System-level strategies

Causal loop	System-level strategies
R1	Evaluation of standardised pre- and post-measures to define success of survivorship programmes which are linked to funding outcomes across private and public settings
	Successful embedment of nutrition and physical activity requires a system that incentivises or encourages general practitioners and hospital systems to assess cancer survivors’ status of nutrition and physical activity. For example, such assessment information needs to be collected and reported and linked to activity-based funding (e.g. blood pressure checks, smoking cessation)
R2	There needs to be coordinated, advocacy efforts to lobby for an increased number of allied health sessions for cancer survivors in terms of dietary and exercise support
	There needs to be system-level and organisation-level efforts to integrate dietary and exercise referrals into existing models of care (i.e. chronic disease management plan, cardiac rehabilitation) as well as developing necessary models of care/funding mechanisms to enable care
R3	Collaborating with universities to recruit students from university placements to improve capacity numbers and future training workforce
	Staff are provided with incentives to specialise, resulting in a more skilled workforce (e.g. increased pay rates, increased job opportunities, reimbursements on successful completion)
	Advocating for nurse practitioners to provide referrals to chronic disease management plans for cancer survivors instead of solely general practitioners, which provides flexibility for general practitioners
R4	Successful optimisation of dietary and exercise services requires information-giving and self-management education, referral for appropriate services, and direct care (from cancer professionals and exercise and nutrition specialists). To facilitate further system optimisation, implementation of a stepped-care model including development of consensus competency frameworks with clear role delineation will be essential
	Development of a competency framework which defines the essential components for role clarification among different health professionals, as well as providing training and resources in relation to this framework
R5	Acknowledging and leveraging on the role of ‘navigation’ or ‘navigators’ to enable integrated systems to facilitate optimal care for all cancer survivors. Cancer survivors should be provided with self-management and practical support to access care so that they know where to go and what they need, to empower them to act
	A centralised, coordinated repository of dietary and exercise services (Exercise & Sports Science Australia, Dietitians Australia, Australian Physiotherapy Association, etc.) in one place with one organisation responsible for collation and maintenance (e.g. Cancer Council, Cancer Australia, Clinical Oncology Society of Australia) is important to promote trustworthiness of information
R6	Closing the loop between health professionals and cancer survivors by sharing medical information through existing digital platforms such as ‘My Health Record’ to ensure clear, timely, and effective bilateral communication processes are adopted
	Technology and information platforms (including information about why cancer survivors should be referred for services, understanding connections between exercise/nutrition and their health outcomes-website, documents) should be co-designed and implemented in partnership with consumers
R7	Development of standard assessment tools that are cancer-specific to assess patient needs and preferences in relation to dietary and exercise support and triage care accordingly. Implementation of these tools can facilitate referrals to dietitians, clinical exercise physiologists, and physiotherapists across multiple settings
	Implementation of standardised screening processes into referral processes (e.g. automated system to screen for care needs and referral pathways, use of electronic patient-reported outcome measures, artificial intelligence, and electronic medical records to flag things automatically)

### Causal loop diagram

**Fig. 2:**
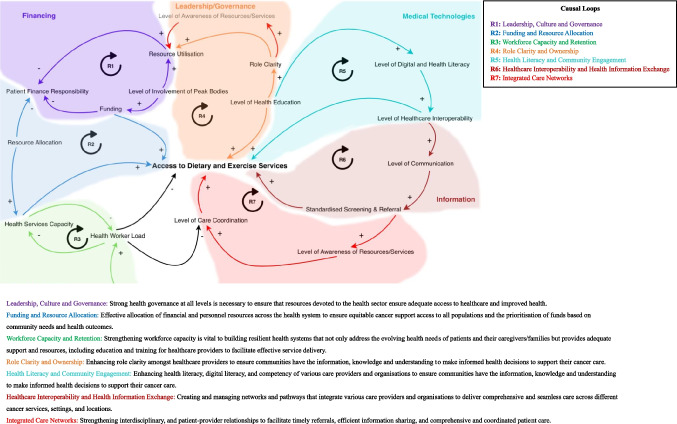
Causal loop diagram

The causal loop diagram represents the causal relationships in the health system based on the relationships identified in this systems-thinking study (Fig. 2). Many (*n* = 7) loops within the diagram were identified, reflecting the complexity of the system. Additionally, each of the loops had an even number of negative links (‘-’) which signified reinforcing feedback loops. A detailed explanation of each feedback loop can be found in Online Resource 4 (Description of causal links and feedback mechanisms).

## Discussion

This systems-thinking study is the first to explore the complex, interconnected relationships between factors and relationships of the health system that influence referral practices for diet and exercise for cancer survivors. Discussions during the workshop from diverse perspectives of consumers, clinicians, not-for-profit organisations, peak professional organisations, and government reaffirmed common priority leverage points as per the causal loop diagram and actions to improve dietary and exercise referral practices. The findings also showcased the potential of collaboration and the development of knowledge across diverse stakeholder groups and of applying complexity and systems-thinking approaches to improve referral practices.

Out-of-pocket costs and insufficient functional funding mechanisms for allied health services were flagged by participants as key barriers influencing access to dietary and exercise services, which can affect cancer survivors’ overall health outcomes [40, 41]. Participants recommended that changes to the financing of allied health services in Australia are required to address the needs of cancer survivors to ensure access to timely and comprehensive dietary and exercise support. For both patients and GPs, having access to subsidised allied health services can be a key factor in starting the care planning process [42]. The current GP Management Plan (GPMP) and Team Care Arrangement items, funded under the Medicare Benefits Schedule [12], allow access to five allied health sessions per year for people with a chronic disease. These five sessions can be shared across all allied health specialities and all disease types and are prioritised by GPs towards certain specialities based on individual care needs. While there has been evidence of the uptake of GPMP allied health service items for people with chronic conditions [42, 43], there has been limited evidence to date for cancer survivors in particular. When exploring the relationship between the use of allied health services and emergency admissions (EA) and potentially preventable hospitalisations (PPH), people with chronic conditions who claimed more physiotherapy sessions (five or more sessions per year) had lower rates of EAs and PPHs than those who did not claim for physiotherapy sessions [42]. Despite this study reaffirming the substantial benefits of physiotherapy services, further research is required to explore other factors influencing claims such as individual needs for additional allied health services, access to outpatient clinics, out-of-pocket costs, and private health insurance. For cancer survivors with coexisting chronic conditions and complex care needs [44], five sessions may be insufficient to meet their individual needs particularly for dietary and exercise services. Without a mechanism for further subsidisation, the system limits people who require but cannot afford allied health services from accessing them. As such, optimal care is only available to those who are able to pay for the additional services required through the private healthcare system [42]. There is a need to determine optimal strategies at different levels (providers, cancer centre, hospital, and government agencies) to address costs of care in order to minimise financial toxicity, promote access to high value care, and reduce health disparities. Future policy efforts should advocate for more funding and alternative financing so that cancer survivors can receive appropriate referrals to the adequate allied health services they require. There needs to be coordinated, advocacy efforts to lobby for an increased number of subsidised sessions for cancer survivors in Australia and streamlining referrals to these sessions. Participants recommended applying a similar level as the Group Allied Health Services for people with type 2 diabetes (i.e. including education, dietary, and exercise interventions) as a reference point for future advocacy efforts [45]. Consequently, government funding and policies are critical to dietary and exercise care provision, so there is a need to leverage existing resources as well as advocating for better access to allied health and support services through adequate funding models to improve patient outcomes.

The Australian Government has spent the last 10 years focusing on primary healthcare reform as a means of addressing the rising expense of healthcare, especially for those with a chronic disease, and the need to focus on system integration. Australia’s Primary Health Care 10 Year Plan 2022–2032 was released by the Australian Government [46] with an agenda outlining short-, medium-, and long-term actions for primary healthcare reform. The Strengthening Medicare Taskforce Report highlighted recommendations to progress the implementation of this 10 Year Plan. These recommendations included funding for longer consultations and the care of chronic disease through hybrid funding approaches combined with fee-for-service to meet patients’ needs, provide affordable care, and enable high-quality, integrated, and person-centred care for all Australians [47, 48]. After the National Cabinet recently approved the taskforce’s recommendations, the Australian Government provided funding for an independent scope of practice review to look into the incentives and challenges faced by health practitioners working to their full scope of practice in primary care [49]. To address these issues and enhance service delivery further, system-wide and focused policy interventions could be developed for chronic disease management, particularly referrals to dietary and exercise services, through effective and functional local health service coordination and sectoral integration.

Breakdowns in networks and poor interprofessional communication pathways were perceived by participants as additional barriers to optimal referrals to dietitians and exercise professionals, as well as communication between healthcare providers and their patients across settings. For example, GPs can often be disconnected from the cancer specialist team due to ineffective communication and poor integration of treatment plans between GPs and cancer specialists [50]. It is well established that poor communication in cancer care is multidimensional and can exert a negative influence on patients’ treatment decisions, symptom management, and quality of life [51]. Integrated systems that use or build upon existing electronic health records (e.g. My Health Record) have the potential to facilitate shared cancer care through improved GP-specialist communication. Early involvement of GPs and two-way communication between acute and primary care throughout the cancer continuum are paramount to ensure optimal patient care, including optimal referral practices. Such communication and intersectoral connection should continually be supported by a capable navigation and digital infrastructure. Even though the Australian healthcare system provides a wide range of services, it is complex to navigate, which can limit the effective connection of cancer survivors with healthcare providers and services that address their individual needs [13, 52]. Effective communication between healthcare providers and patients is a necessity to meet patient needs and to provide high-quality services such as ensuring that patients are aware about the existing dietary and exercise resources/services that they can access (e.g. GPMP [12], Optimal Care Pathways [53], Cancer Council online resources [54]). In addition to better communication, the roles of healthcare providers in the cancer care continuum must be identified and defined, taking into account the essential contributions of GPs, cancer specialists, and allied health professionals and avoiding significant overlap in important cancer care provision, contributing to better care coordination [8]. Delivering coordinated and person-centred care could be improved by adopting standardised screening and referral processes, and using screening tools at key transition points to identify patients’ needs and streamline referrals to dietitians and exercise professionals. Adoption of healthcare interoperability across different settings can help promote continuity and clarity within the patient care team and patient-centred care.

Participants also highlighted that there needs to be a more integrated and coordinated approach between peak bodies and accredited bodies such as the Australian Physiotherapy Association (APA), Exercise and Sports Science Australia (ESSA), Dietitians Australia (DA), Clinical Oncology Society of Australia (COSA), Cancer Councils, Nutrition Australia, Fitness Australia, care providers, GPs, local health networks (LHNs), and leadership mentoring systems. Many of these organisations play an important role in providing national leadership and fostering improvements in the integration of networked cancer services. One such example includes the development of the five principles underpinning Australia’s primary care response to COVID-19 [55]. Together with representatives from primary healthcare and more than 30 peak national organisations, these principles were quickly formulated to guide policy at the outset of the pandemic [55]. Effective collaboration between these organisations during COVID-19 underscores the importance of integrating and coordinating care among separate care entities. Due to challenges in integrating and coordinating care across various healthcare services [56], one way to better integrate care may involve bringing siloed services together through the development of a centralised repository of dietary and exercise services with one organisation responsible for collation and maintenance. Peak bodies and accredited organisations can each undertake key roles such as coordinating the development of information about the available cancer services in each state and territory for GPs and cancer survivors. This information could potentially benefit both providers and cancer survivors as GPs are supported to have optimal referral practices and cancer survivors are reassured of the best system-based care.

This study is the first to use a systems-thinking approach with a complexity lens to explore referral practices in cancer care. The use of a complex system mapping process and the WHO building blocks framework is especially important in cancer survivorship for exploring the various interactions within the healthcare system across the cancer care continuum.

This study has some limitations. Given the qualitative nature, perspectives of the participants might be subjected to their experiences and perspectives. However, the workshop participants represented a range of diverse perspectives including experts in the fields (i.e. researchers, healthcare professionals, consumers, and policy makers). Despite efforts to recruit and include GPs, GPs who were invited were not able to engage. Therefore, their perspectives were underrepresented in this study due to challenges in recruitment. Furthermore, although cancer survivors were included in this study, greater representation is required as their interactions with the system are important perspectives. Overall, this study provided outputs that have the potential to inform implementation and strengthen health systems at various levels in terms of dietary and exercise referral practices.

## Conclusion

Adopting a systems-thinking approach with a complexity lens enabled health professionals, service providers, and policy makers to identify the complex interplay of factors and touchpoints influencing dietary and exercise referral practices. Strategies identified from this study can be used to change the direction of causal loops in the causal loop diagram which can directly or indirectly influence other system-level factors. The causal loop diagram developed in this study has the potential to inform local action plans for implementation using identified strategies to address leverage points and for mitigating possible future risks in the system. These findings can be tailored to inform other healthcare systems and international efforts to meet the needs of cancer survivors in terms of dietary and exercise support. Future research could translate these strategies into actions and evaluate the implementation of essential elements of dietary and exercise referral practices in practice as informed by this causal loop diagram and system-level strategies.

### Supplementary Information

Below is the link to the electronic supplementary material.

Supplementary file1 (PDF 76 KB)

Supplementary file2 (PDF 32 KB)

Supplementary file3 (PDF 142 KB)

Supplementary file4 (PDF 114 KB)
