# Supplementary figures and images for: Adopting a systems-thinking approach to optimise dietary and exercise referral practices for cancer survivors
Source: Support Care Cancer. 2024 Jul 10;32(8):502. doi: 10.1007/s00520-024-08692-z (PMC11236908; doi:10.1007/s00520-024-08692-z)

## WHO building blocks

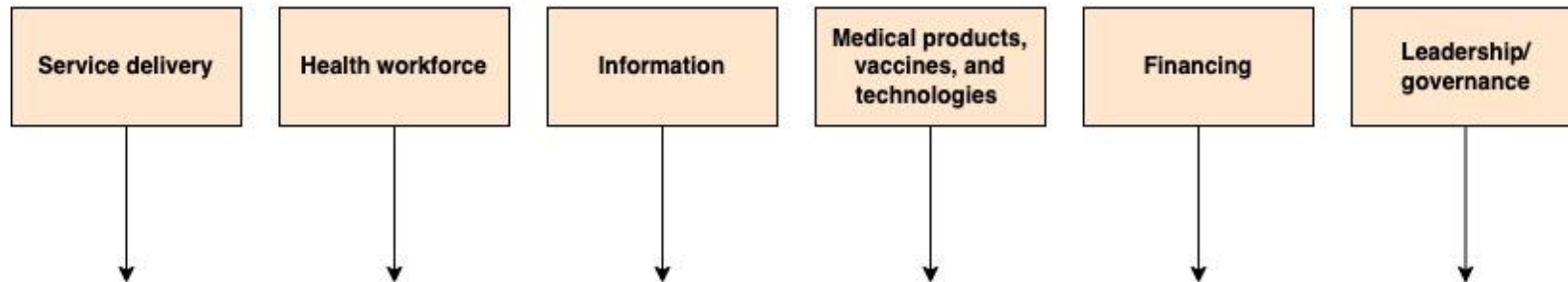

## Priorities

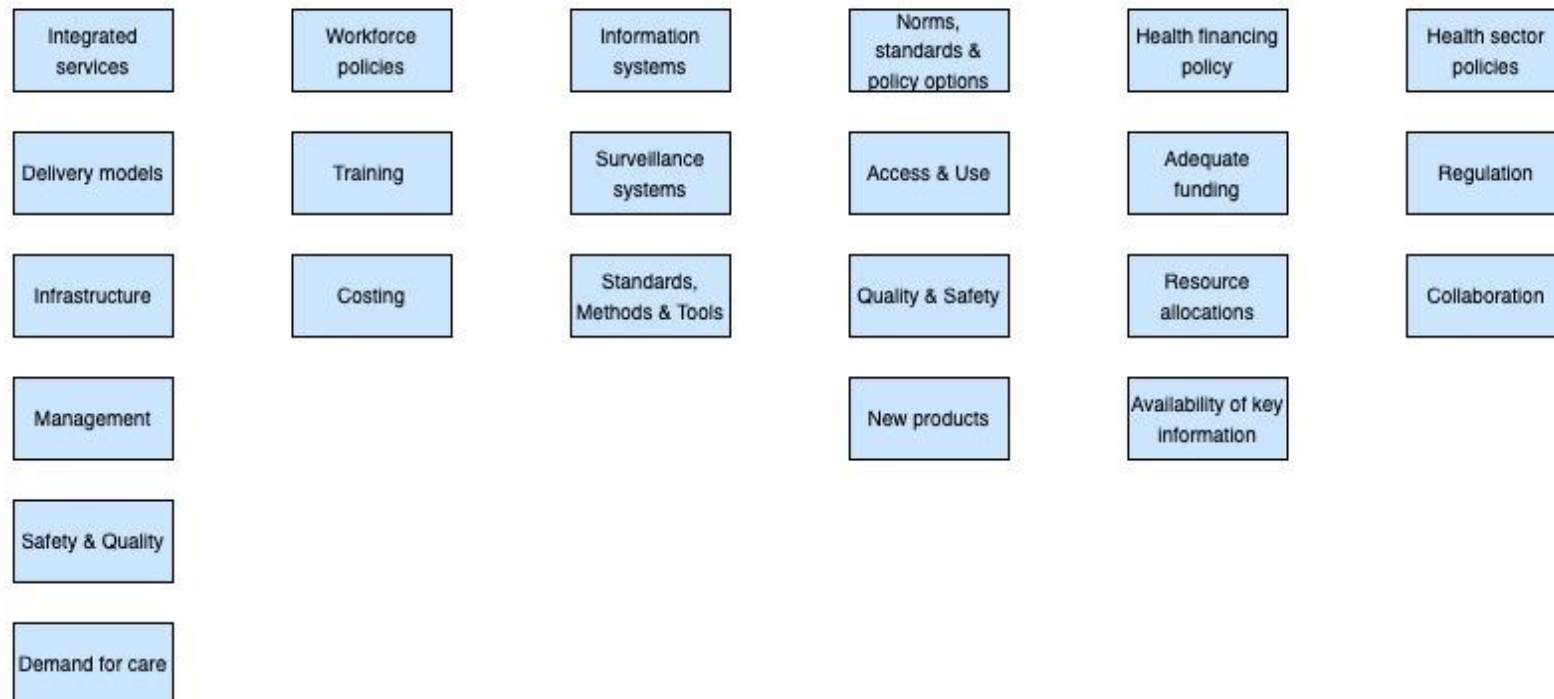

Supplement: Supplementary file 1 — Supplementary file1 (PDF 76 KB) [file 520_2024_8692_MOESM1_ESM.pdf]

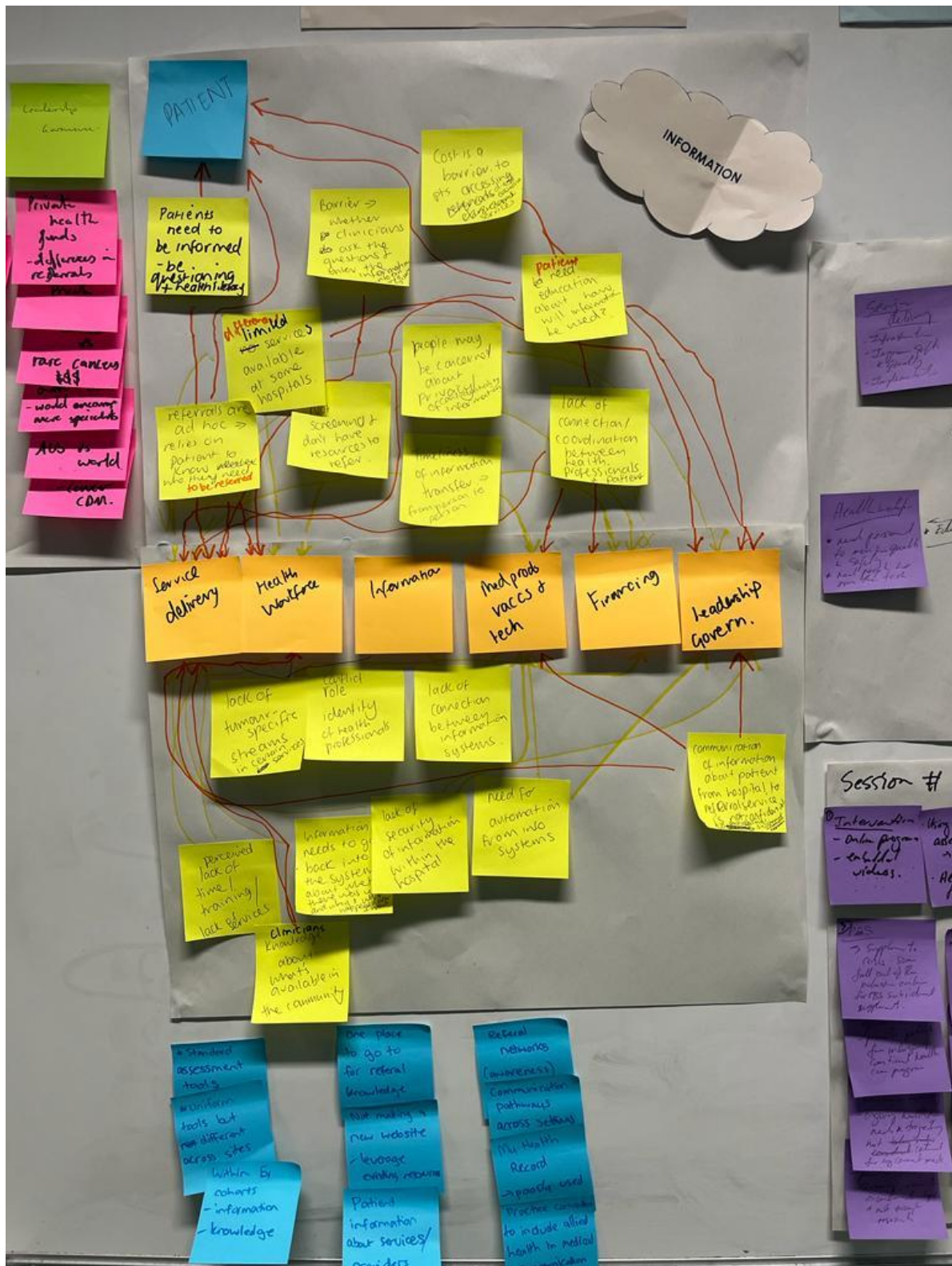

Supplement: Supplementary file 3 — Supplementary file3 (PDF 142 KB) [file 520_2024_8692_MOESM3_ESM.pdf]
