# Supplementary material for: Adopting a systems-thinking approach to optimise dietary and exercise referral practices for cancer survivors
Source: Support Care Cancer. 2024 Jul 10;32(8):502. doi: 10.1007/s00520-024-08692-z (PMC11236908; doi:10.1007/s00520-024-08692-z)
Supplement: Supplementary file 2 — Supplementary file2 (PDF 32 KB) [file 520_2024_8692_MOESM2_ESM.pdf]

Category	Barriers/Facilitators	
**Financing**	System-level	• Resource allocation (e.g., grant funded services)<br>• Lack of funding (e.g., federal vs state, state by state differences, private vs public system)<br>• Activity based funding (ABF) in hospitals<br>• MBS item billing (CDMP)<br>• No clear funding pathway<br>• Costs for common cancers vs rare cancers
	Patient-level	• Out of pocket costs for patient/patient finances
**Service Delivery**	System-level	• Infrequent and inconsistent screening practices by health professionals<br>• Lack of advocacy for scope of practice<br>• Inadequate use of guidelines and standards for measures and output<br>• Lack of evaluation of data for Care MDT<br>• Implementation of Optimal Care Pathways<br>• Ad-hoc referrals (relies on patient knowledge re services)<br>• Insufficient access to allied health support (i.e., 5 sessions per year)<br>• Cost barriers<br>• Geographic barriers<br>• Lack of tumor specific streams in certain services
**Information**	System-level	• Lack of communication pathways between health professionals and patients across settings.<br>• Lack of accreditation standards for outpatient/community compared to inpatients<br>• Limited access to medical records for private providers<br>• Lack of security of information within the hospital (e.g., patient concerns about privacy/confidentiality of information)
	Provider-level	• Lack of training and CPD<br>• Conflict role identity of health professionals<br>• Lack of awareness of resources/services.<br>• Level of experience regarding knowledge of whether patient needs physiotherapist or exercise physiotherapist<br>• Lack of community expertise for regional health
	Patient-level	• Lack of awareness of resources/services.<br>• Lack of digital and health literacy<br>• Trust and reliability of information for patients
**Leadership/Governance**	System-level	• Fragmented leadership/responsibilities involving peak bodies and accredited bodies such as APA, ESSA, DA, COSA, Nutrition Australia, Fitness Australia, cancer councils, care providers, GPs, LHNs, leadership mentoring systems<br>• Lack of involvement of all stakeholders from the beginning resulting in a fragmented system<br>• Lack of pathway/chain (e.g., Cancer Australia plan can be implemented into services)<br>• Barriers in care coordination (e.g., My Health record is poorly used).<br>• Lack of guidance for current clinical system<br>• Lack of structure for cancer type differences
**Technologies**	System-level	• Technology limitations (e.g., data breaches)<br>• Lack of connection between information systems<br>• Technologies not supported by healthcare system/connectivity issues
**Health Workforce**	System-level	• Limited staff capacity/services<br>• Lack of personnel to manage quality and safety<br>• Resource allocation to different cancer types (some cancers get allocated more resources than others)<br>• Lack of work-from-home resources

		• Limited services available at some hospitals<br>• Leveraging existing resources such as my health record
	Provider-level	• Time constraints<br>• Reluctance to refer patients from acute care due to reduced trust<br>• Reluctance to own care<br>• Risk-adverse<br>• Lack of commitment<br>• Contribution of students during their placements
	Patient-level	• Patient demand
