# Supplementary material for: Adopting a systems-thinking approach to optimise dietary and exercise referral practices for cancer survivors
Source: Support Care Cancer. 2024 Jul 10;32(8):502. doi: 10.1007/s00520-024-08692-z (PMC11236908; doi:10.1007/s00520-024-08692-z)
Supplement: Supplementary file 4 — Supplementary file4 (PDF 114 KB) [file 520_2024_8692_MOESM4_ESM.pdf]

Description of causal links and feedback loops	
**R1: Leadership, Culture and Governance**<br>Greater involvement of peak bodies may influence funding and resource utilisation. An increase in funding and resource utilisation may decrease patient financial responsibility. Increased awareness of resources/services may also result in greater utilisation of resources.	Reinforcing feedback loop
**R2: Funding and Resource Allocation**<br>Effective resource allocation may increase health service capacity as well as influencing patient financial responsibility.	Reinforcing feedback loop
**R3: Workforce Capacity and Retention**<br>Increased patient demand may increase health worker load and reduce health services capacity.	Reinforcing feedback loop
**R4: Role Clarity and Ownership**<br>Increases in health education may improve the clarity of role responsibilities among interprofessional team members, which may promote awareness of existing resources/services that are available in terms of dietary and exercise support. Increased awareness of resources/services may also influence the utilisation of resources.	Reinforcing feedback loop
**R5: Health Literacy and Community Engagement**<br>Increases in health education may improve digital and health literacy, which may increase healthcare interoperability across healthcare settings.	Reinforcing feedback loop
**R6: Healthcare Interoperability and Health Information Exchange**<br>Increased healthcare interoperability across healthcare settings may improve communication levels between healthcare organisations, healthcare providers and patients, which may improve standardised screening and referral practices.	Reinforcing feedback loop
**R7: Integrated Care Networks**<br>An increase in standardised screening and referral practices may increase the level of awareness regarding resources/services that are available in terms of dietary and exercise support, which may support care coordination.	Reinforcing feedback loop
